# Radiological Outcomes of Magnetically Controlled Growing Rods for the Treatment of Children with Various Etiologies of Early-Onset Scoliosis—A Multicenter Study

**DOI:** 10.3390/jcm13061529

**Published:** 2024-03-07

**Authors:** Pawel Grabala, Munish C. Gupta, Daniel E. Pereira, Michal Latalski, Anna Danielewicz, Pawel Glowka, Michal Grabala

**Affiliations:** 1 Department of Pediatric Orthopedic Surgery and Traumatology, University Children’s Hospital, Medical University of Bialystok, Waszyngtona 17, 15-274 Bialystok, Poland; 2 Paley European Institute, Al. Rzeczypospolitej 1, 02-972 Warsaw, Poland; 3 Department of Orthopaedic Surgery, Washington University in St. Louis, 660 S Euclid Ave, St. Louis, MO 63110, USA; munishgupta@wustl.edu (M.C.G.); d.e.pereira@wustl.edu (D.E.P.); 4 Paediatric Orthopaedic Department, Medical University of Lublin, Gebali 6, 20-093 Lublin, Poland; michallatalski@umlub.pl (M.L.); anna.danielewicz@umlub.pl (A.D.); 5 Department of Spine Disorders and Pediatric Orthopaedics, Poznań University of Medical Sciences, 28 Czerwca 1956 r. Street, no. 135/147, 61-545 Poznań, Poland; pawel.glowka@ump.edu.pl; 6 2nd Clinical Department of General and Gastroenterogical Surgery, The Medical University of Bialystok Clinical Hospital, Medical University of Bialystok, M. Skłodowskiej-Curie 24a, 15-276 Bialystok, Poland; michal@grabala.pl

**Keywords:** early-onset scoliosis, EOS, magnetically controlled growing rods, MCGR, pediatric spinal deformity, growing rods, juvenile scoliosis

## Abstract

**Background:** The management of spinal deformities diagnosed before the age of 10 is critical due to the child’s development, skeletal system, and growth mechanism. Magnetically controlled growing rods (MCGRs) are a surgical treatment option for the growing spine. The aim of this study was to analyze the radiological findings of patients treated with MCGRs for early-onset scoliosis (EOS) of various etiologies. We hypothesized that the MCGRs could provide acceptable long-term radiographic results, such as an increase in the T1–T12 and T1–S1 height and significant overall deformity correction. **Methods:** We retrospectively reviewed 161 EOS patients with a combined total of 302 MCGRs inserted at five institutions between 2016 and 2022 with a mean follow-up of at least two years. The Cobb angle of the major curve (MC), thoracic kyphosis (TK), lumbar lordosis (LL), and T1–T12 and T1–S1 height measurements were assessed before, after, and during the follow-up. **Results:** Among the 90 female and 71 male patients, there were 51 neurological, 42 syndromic, 58 idiopathic, and ten congenital scoliosis etiologies. Of the patients, 73 were aged under six years old. The mean follow-up time was 32.8 months. The mean age at placement of the MCGRs was 7 years and that at the last follow-up after fusion surgery was 14.5 years. The mean MC before the initial surgery was 86.2°; following rod implantation, it was 46.9°, and at the last follow-up visit, it was 45.8°. The mean correction rate among the etiology subgroups was from 43% to 50% at follow-up. The mean TK was noted as 47.2° before MCGR implantation, 47.1° after MCGR placement, and 44.5° at the last follow-up visit. The mean T1–T12 height increased by 5.95 mm per year, with a mean T1–S1 height of 10.1 mm per year. **Conclusions:** MCGR treatment allowed for an average correction of the curvature by 50% during the period of lengthening, while controlling any deformity and growth of the spine, with a significant increase in the T1–T12 and T1–S1 values during the observation period. MCGR treatment in EOS carries a risk of complications. While congenital and syndromic EOS often have short and less flexible curves in those groups of patients, single rods can be as effective and safe. Definitive fusion results in the mean final coronal correction between the start of MCGR treatment and after undergoing PSF of approximately 70%. The mean T1–T12 spinal height increased by 75 mm, while the T1–S1 spinal height gained a mean of 97 mm.

## 1. Introduction

Spinal deformities occurring in children and adolescents, due to their growth potential and developmental age, are most accessible to divide into those that appear over the age of 10 and those that are diagnosed and require treatment before the age of 10 [1]. Curvatures diagnosed before the age of 10 are extremely important as they contribute to the child’s development, skeletal system, and growth mechanism, as well as the development of the lungs, chest, and internal organs. In addition to differentiating these curves in terms of the child’s age, there is also an etiological element, i.e., congenital, neuromuscular, syndromic, or idiopathic, that is usually present [2]. Thus, undertaking the necessary treatment is extremely important in children under ten years of age in preventing long-term sequela from a severe spinal deformity. The decision to start surgical treatment for early onset scoliosis (EOS) is difficult as a balance must be maintained between the patient’s age, stabilization of deformities, and the growth of the spine and chest, which translates into lung development and respiratory capacity [3,4,5,6].

In the surgical treatment of spinal deformities in children up to 10 years of age, various surgical techniques and implants are used to promote growth [7,8,9,10,11]. Developed in 2009, magnetically controlled growing rods (MCGRs) were intended to improve treatment and minimize the risk of complications, and especially to reduce the number of repeated surgeries and anesthesia performed to extend the instrumentation for standard growing rods [11,12,13,14,15,16]. The surgical technique itself is minimally invasive, requiring only two mini-incisions in the upper and lower spine, and the rods are attached submuscularly or subfascially to the spine using pedicle screws or hooks to connect the proximal and distal bases and placed rods [17,18]. After the initial surgery with the insertion of the MCGRs, rod distraction is performed without anesthesia, using only special equipment, i.e., an external remote control (ERC), without repeated operations. However, the procedure is not without its complications, with a significant risk of unplanned surgery, and concerns have been raised regarding the structural integrity of MCGR implants [12,14,19,20,21]. Most published studies looking at MCGR include small numbers of patients and short follow-up times.

The aim of this multicenter study was to collect and analyze the radiological findings of patients treated with MCGRs for EOS of various etiologies. We hypothesized that MCGRs could be effective over time and bring up long-term radiographic results beyond 2 years after the initial surgery.

## 2. Materials and Methods

### 2.1. Setting and Patients

This consecutive series included all patients treated with MCGRs for EOS at five institutions between 2016 and 2022. Patients were identified via local surgical records. The treatment for all patients was fully financed within the public healthcare system. This study was conducted in accordance with the Declaration of Helsinki (as revised in 2013). After obtaining the approval of the regional Bioethics Committee for this study, we conducted a thorough analysis of the documentation of patients treated for pediatric and early childhood scoliosis. We retrospectively reviewed all EOS patients with MCGRs and a mean follow-up of at least two years (unless revision surgery was performed). Patients with previous spinal surgeries using instruments other than MCGRs were excluded.

**Figure 1:**
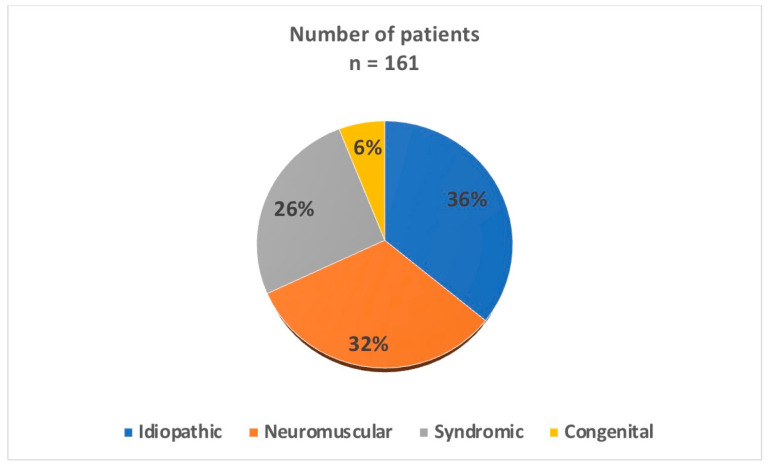
Patient demographics based on etiology.

One hundred and sixty-one patients met the inclusion criteria, resulting in a total of 302 MCGRs implanted during treatment. Patients treated surgically for spinal deformities of various etiologies during growth were classified according to the EOS classification [22] (Figure 1).

### 2.2. Outcome Parameters

We extracted radiological measurements before the surgical treatment, before the insertion of magnetic rods, immediately after their implantation, and during the observation period. Before the start of treatment, each patient underwent magnetic resonance imaging of the entire spine in order to exclude other diseases within the spinal cord and spine. Measurements of the Cobb angle of the major curvature (MC), thoracic kyphosis (TK), lumbar lordosis (LL), and T1–T12 and T1–S1 height measurements were assessed before, after, and during the follow-up each time. For the kyphosis and lordosis angle measurements, the superior endplate of the upper-end vertebrae and the inferior endplate of the lower-end vertebra were utilized. The correction obtained in the postoperative period and during the period of observation of the MC angle (measured by the Cobb method) was expressed as the ratio of the preoperative angle of MC in the coronal plane minus the postoperative angle of MC in the coronal plane divided by the preoperative angle of MC. Proximal junction kyphosis (PJK) was defined radiographically as a change in the kyphosis angle > 10° above the upper two levels of the vertebrae compared with the first erect postoperative image. The T1–T12 and T1–S1 heights were measured as described by Cheung et al. [8,16]. For the measurement of thoracic kyphosis, T1–T12 height, and T1–S1 height in cases with an abnormal number of thoracic vertebrae, the inferior endplate on the most distal thoracic vertebra (defined by the existence of at least one rib) was used as the distal measuring point. Growth in the T1–T12 and T1–S1 segments after MCGR implantation was calculated as the latest postoperative value minus the first postoperative value. The postoperative growth rate was calculated by dividing the growth value by the time elapsed between the first and latest postoperative value. The annual postoperative growth rate of the T1–T12 and T1–S1 segments was calculated in patients with a minimum postoperative radiographic follow-up of 1 year to avoid overestimation. Instrumentation levels, anchor type (pedicle screw or hook), and complications such as infection, anchor pull-out, rod breakage, pin fracture, distraction failure, adding-on, and PJK were recorded. The number of rod exchanges was also recorded. Revision procedures and definitive surgeries that were performed ahead of schedule due to complications were labeled as unplanned.

### 2.3. Surgical Technique and Postoperative Use of MCGRs

Our surgical technique was a less-invasive approach to the spine with two short incisions, one on the top and one on the bottom spine, with segmental screws and hooks used on the top, segmental screws put on the bottom, and subfascial insertion of MCGRs as described by Grabala et al. [17] and Chamberlain at al. [18]. Our postoperative distraction protocol for MCGRs was similar to other cases of postoperative lengthening described in the literature [17,23,24,25] and was adapted for our patients. For all patients, we ordered a brace for three months. After this period, we started lengthening with an external remote controller (ERC). For most patients, we performed distractions every eight weeks and lengthened the MCGRs by 2–2.5 mm depending on the growth potential. We performed control X-rays every six months or when the patients complained of new symptoms, like back pain, implant prominence, and neurological deficits, or when we noted any suspicious loosening of implants or rod fractures during physical examination. We analyzed the X-rays for implant fractures, screw loosening, assessment of the length of rod extension, evaluation of growth parameters for the length of the spine, and assessment of curvature parameters in the sagittal and coronal planes.

### 2.4. Complications

We adopted the following definitions for this study: All adverse events and complications were divided into early (revealed in the period from the day of surgery to 3 months after surgery) and late (revealed later than three months from the original surgery). Patients qualified for revision surgery if they experienced an adverse event or complication that could not be treated conservatively, that threatened the safety and health of the patient, and that required prompt surgical intervention. Patients who were treated with MCGRs and experienced complications such as rod fracture and PJK development or reached the end of rod lengthening, skeletal maturity, or the minimum parameters T1–T12 and T1–S1 for conversion to PSF were operated electively [12,21,26]. In this study, we only noted the number of complications and the revision rate because all complications and unplanned surgeries have been analyzed very carefully in other studies.

### 2.5. Statistical Analysis

SPSS Statistics (SPSS) v. 27 (IBM Corp., Armonk, NY, USA) was used for all statistical analyses. Before analysis, the data were tested for normality using the Z-values of skewness and kurtosis, histograms, and Q–Q plots. The normal distribution was analyzed by the Shapiro–Wilk test. Student’s *t*-test and ANOVA (analysis of variance) were used for quantitative variables and the chi-square test for qualitative variables. Pearson’s correlation coefficient was used to assess the relationship between the radiographic follow-up time and the T1–T12 and T1–S1 height. If not otherwise specified, the data are presented as the mean ± standard deviation. Significance was set at *p* < 0.05 for all analyses. STROBE guidelines were adopted for the reporting of the results.

## 3. Results

### 3.1. Patient Characteristics

Among the 161 patients (90 females and 71 males), there were 51 neurological (NS) (32%), 42 syndromic (SS) (26%), 58 idiopathic (IS) (36%), and ten congenital (CS) (6%) scoliosis etiologies, as presented in Figure 1. Of the patients, 73 (45%) were under 6 years of age, while the other 88 patients (55%) were over six years of age. The mean follow-up time was 32.8 months (12–68 months). The mean age at placement of the MCGRs was 7.08 years (range: 2.5–14 years), and that at the last follow-up after removing the MCGRs and undergoing fusion surgery was 14.5 years (range: 11–16 years) (Table 1).

**Figure 2:**
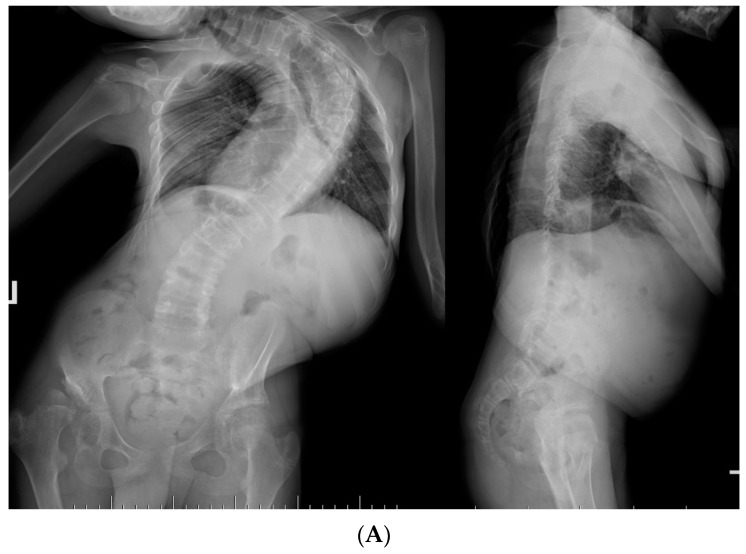

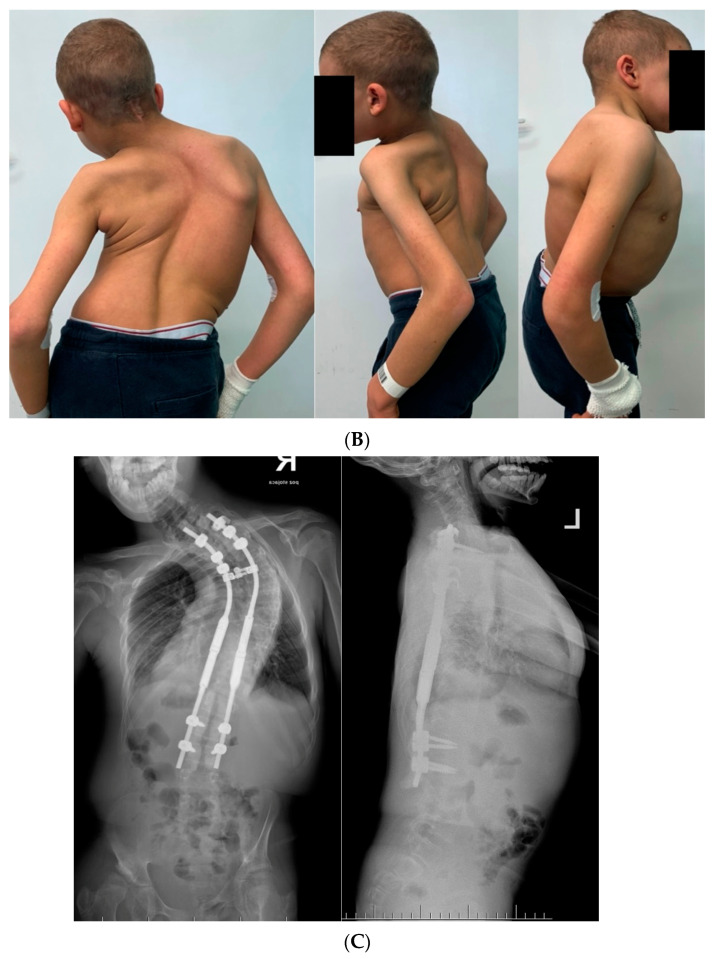

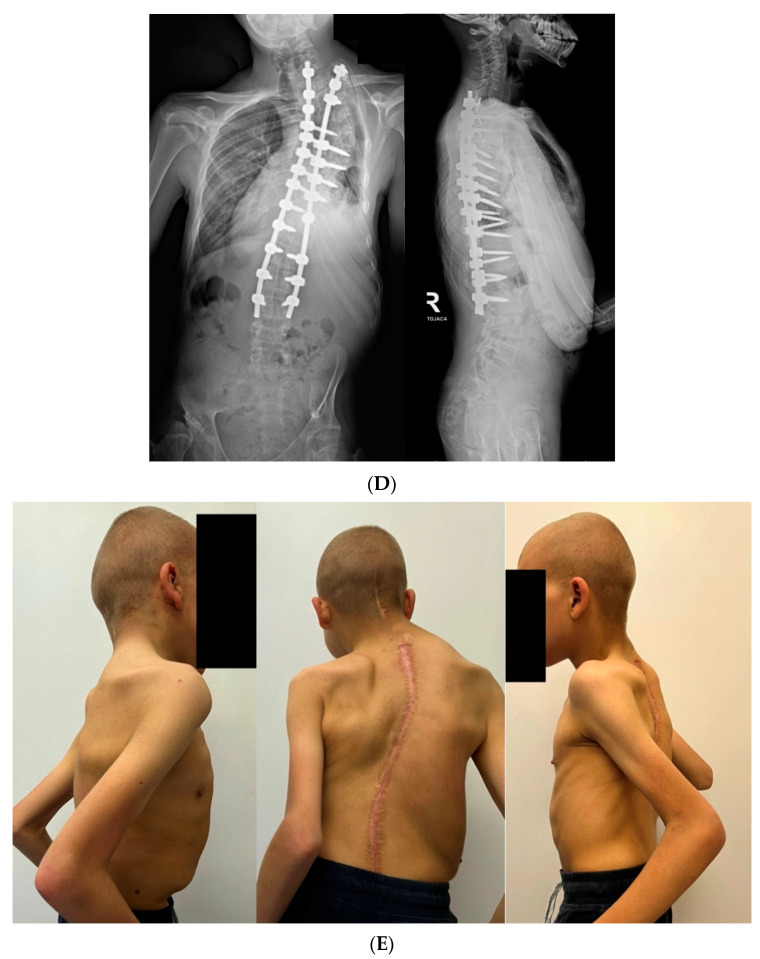
Fourteen-year-old immature patient with a rare case of Costello syndrome (**A**,**B**), treated initially with MCGRs (**C**) and converted to posterior spinal fusion after three years of periodic MCGR lengthening (**D**,**E**).

One patient benefited from a late lengthening program because he delayed bone maturation due to his underlying pathology (Costello syndrome), which allowed for a gain in thoracic height. For this patient, the lengthening period ended at 17 years of age and conversion to PSF (Figure 2).

For all of the analyzed patients, 66 (22%) 4.5 and 5.0 mm rods were inserted, while the remaining 236 (78%) rods that were inserted were 5.5 and 6.0 mm in diameter. A total of 114 rods (38%) were 70 mm in length, while the other 188 rods (62%) were 90 mm in length. Twenty patients (12.5%) received single-rod constructs, while the other 141 patients (87.5%) had double-rod constructs inserted. In total, 66 patients (41%) had severe scoliosis of more than 90°, while the other 95 patients (59%) had a main curve of less than 90°. Preoperative HGT was used for 32 patients (20%), and 16 patients (10%) underwent anterior release. Those patients who underwent anterior release did not receive preoperative HGT. All these data are presented in Table 1.

### 3.2. Radiological Measurements

**Figure 3:**
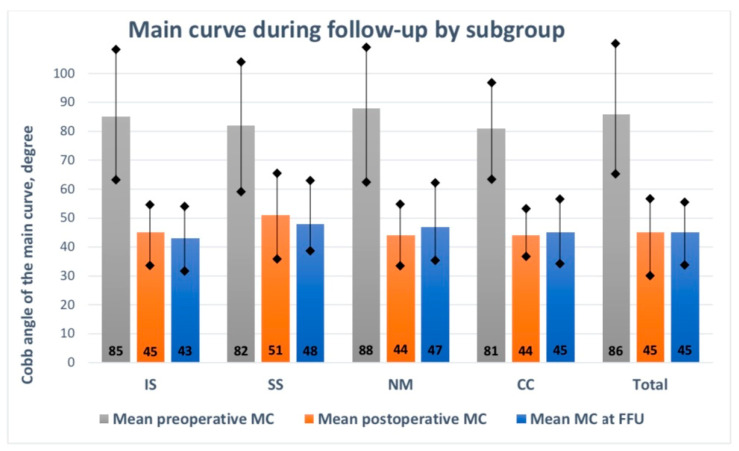
Main curve by subgroup during follow-up. IS—idiopathic scoliosis, SS—syndromic scoliosis, NM—neuro-muscular scoliosis, CC—congenital scoliosis.

For all the analyzed patients, before MCGR implantation, the mean MC was 86.2° (range: 65–122°). Following rod implantation, it was 46.9° (range: 13–81°), while it was 45.8° (range: 9–82°) at the last follow-up visit (*p* < 0.001). The mean correction rate among the subgroups was from 43% to 50% at follow-up (*p* < 0.001), as presented in Figure 3.

**Figure 4:**
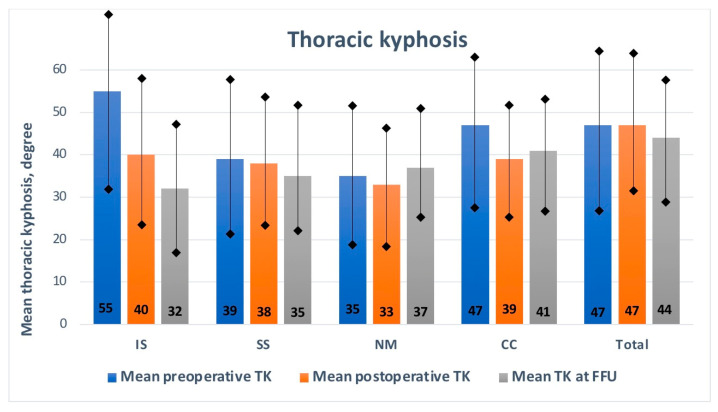
Thoracic kyphosis by subgroup during follow-up. IS—idiopathic scoliosis, SS—syndromic scoliosis, NM—neuro-muscular scoliosis, CC—congenital scoliosis.

The mean TK was 47.2° (range: 18–105°) before MCGR implantation, 47.1° (range: 32–61°) after MCGR implantation, and then 44.5° (range: 8–72°) at the last follow-up visit, with a significant difference (*p* < 0.001) among the subgroups in terms of the pre- and postoperative values, as presented in Figure 4 and Table 2.

LL significantly decreased post-implantation (first erect) but then increased during lengthening. The mean preoperative lumbar lordosis was 44.2° (range: 26–64°), after MCGR placement was 39° (range: 30–54°), and at the last follow-up visit was 45.8° (range: 19–68°). There were no differences between the HGT and non-HGT groups or between age groups (under and over 6 years of age). The T1–T12 thoracic height increased from a mean of 166 mm (range: 92–248 mm) at baseline to 188 mm (range: 118–272 mm) after MCGR implantation, and then to 208 mm (range: 148–282) mm at the last follow-up visit (*p* < 0.001). The T1–S1 height increased from a mean of 295 mm (range: 169–382 mm) at baseline to 328 mm (range: 204–408 mm) after MCGR implantation, and then to 368 mm (range: 241–441 mm) at the last follow-up visit (*p* < 0.001), as shown in Table 3.

### 3.3. Lengthening Period and Final Fusion

The distraction period lasted an average of 37.5 months (18–58 months). The mean number of times a patient underwent lengthening per year was 6.7 (range: 4–9), with a mean T1–T12 lengthening of 5.95 mm (range: 3–8 mm) and a mean T1–S1 lengthening of 10.1 mm (range: 3–16 mm) per year. The mean time from MCGR insertion to the first lengthening was 14 weeks (range: 8–16 weeks), while the mean interval between lengthening was 8 weeks (range: 6–14 weeks), as presented in Table 4.

On average, the number of surgeries per patient (including anterior release, conversion to PSF, and revisions) over the entire follow-up period was 1.6 (range: 2–5). The mean average rod length gain at the final follow-up was 22.16 mm (range: 8–46.3 mm). The mean average rod length gain at PSF was 29.38 mm (range: 11–46.3 mm). No difference was seen among the subtypes of EOS in terms of rod length gain. The mean final coronal correction between the start of MCGR treatment and after undergoing PSF was 70%. The T1–T12 spinal height increased by a mean of 75 mm, while the T1–S1 spinal height gained a mean of 97 mm.

### 3.4. Analysis of the Subgroups

The mean preoperative MC values in all subgroups due to the etiology of the spinal deformity significantly improved by the final follow-up visit (*p* < 0.001), as presented in Figure 2 and Table 2. The mean follow-up period was similar, without any statistically significant difference (*p* > 0.05). Postoperative comparisons of the mean major curve values during the follow-up period showed similar results for all subgroups (N.S.). The mean preoperative thoracic kyphosis values in all subgroups due to the etiology of the spinal deformity significantly improved by the final follow-up visit (*p* < 0.001), as presented in Figure 3 and Table 2. Postoperative comparisons of the mean thoracic kyphosis values during the follow-up period showed significant differences in the IS, NS, and CC subgroups (*p* < 0.001). There were no differences between preoperative and postoperative lumbar lordosis during the follow-up period in any subgroups (N.S.). Still, we noted statistically significant differences among the values during the follow-up period for the IS, CC, and SS groups (*p* < 0.001). The mean preoperative T1–T12 height increased significantly by the last follow-up visit (*p* < 0.001). The most significant value was observed for the IS subgroup. A significant difference in T1–T12 height gain was noted among the subgroups, with the highest value (6.2 mm/year) received in the idiopathic group vs. 5.2 mm/year in the congenital group, 5.5 mm/year in the neuromuscular group, and 5.8 mm/year in the syndromic group (*p* < 0.001), as shown in Table 5.

**Figure 5:**
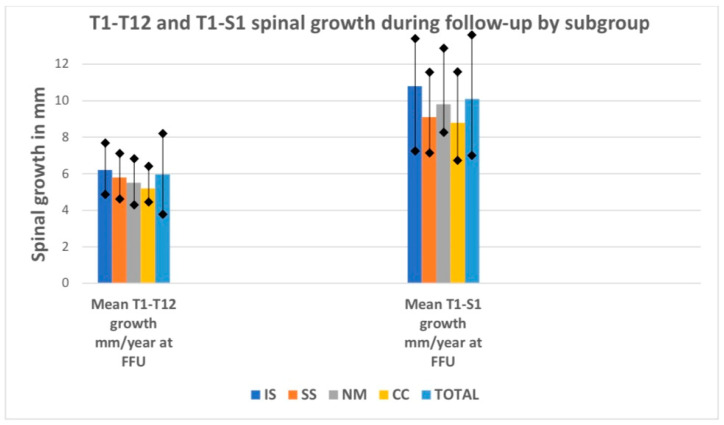
T1–T12 and T1–S1 spinal growth during the follow-up period by subgroup (in mm/year). IS—idiopathic scoliosis, SS—syndromic scoliosis, NM—neuro-muscular scoliosis, CC—congenital scoliosis.

**Figure 6:**
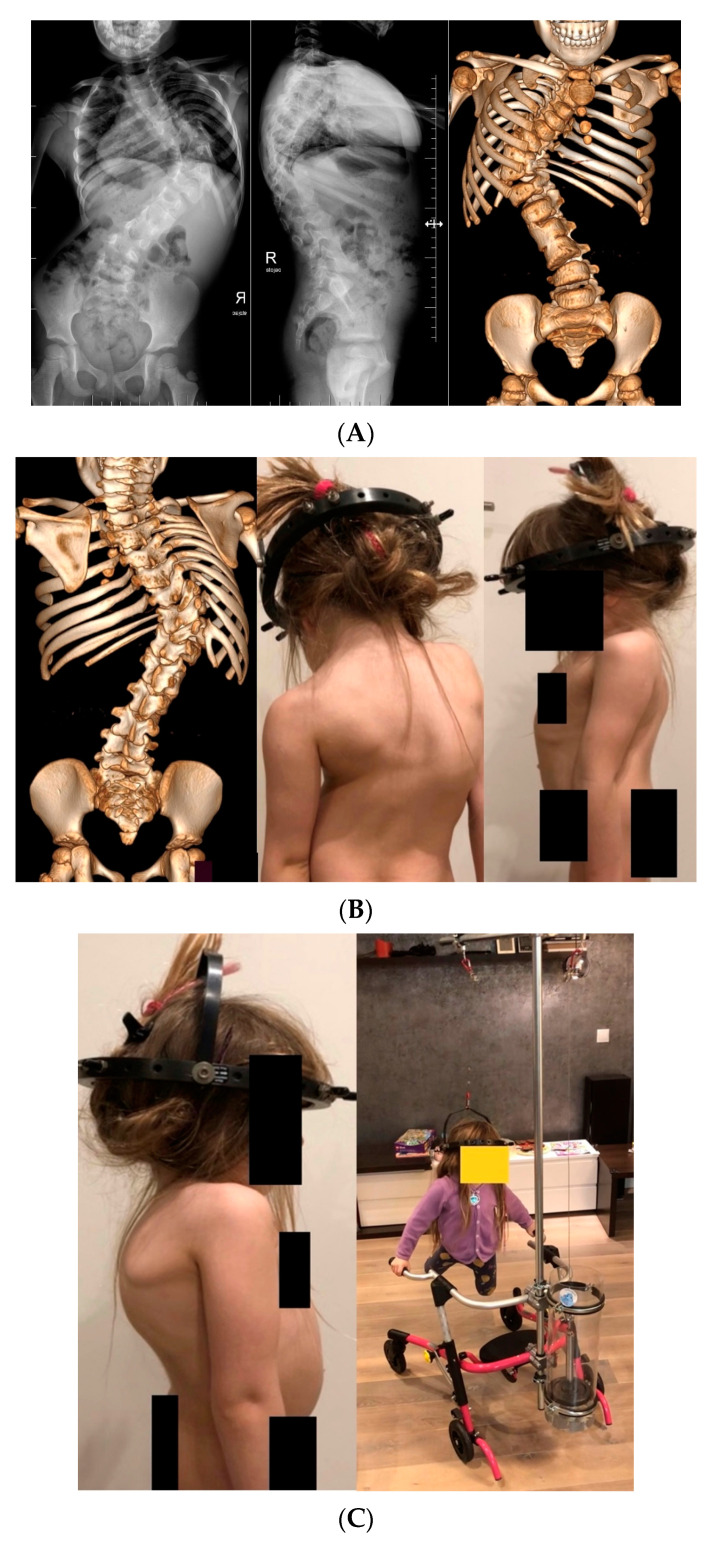

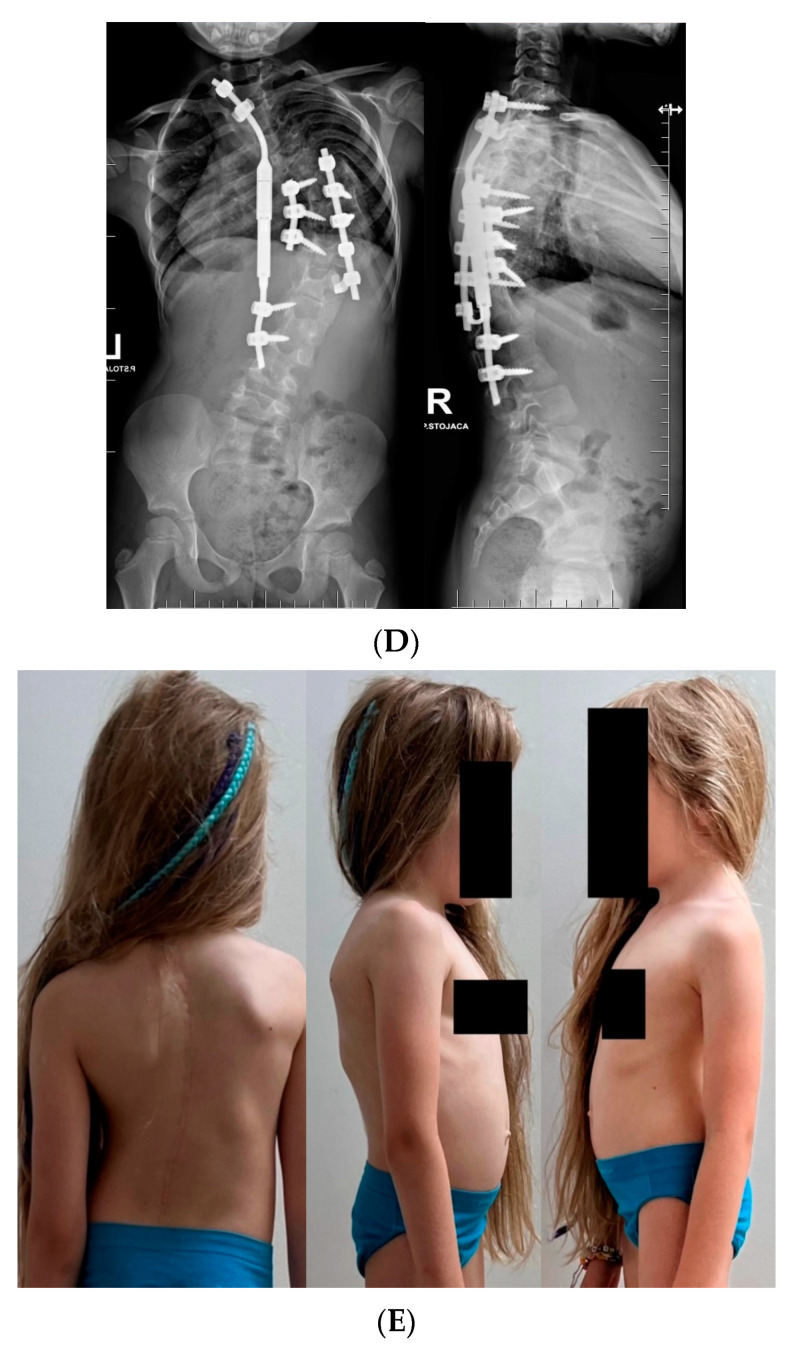
Five-year-old girl with severe congenital scoliosis (multilevel hemivertebrae and blocked vertebrae) reaching 120° (**A**,**B**). A preoperative HGT was used for 3 months (**C**), as well as two levels of P-VCR with short fusion and a single-MCGR construct for distraction left side. X-rays (**D**) and clinical pictures before the treatment course and after the 2-year follow-up period (**E**).

There was a difference in the T1–S1 height gain among the subgroups, with a mean of 10.8 mm/year received in the idiopathic group vs. 9.1 mm/year in the syndromic group, 9.8 mm/year in the neuromuscular group, and 8.8 mm/year in the congenital group (*p* < 0.001), as shown Figure 5. Figure 6 presents a case of severe congenital scoliosis in a 5-year-old girl treated with P-VCR and a single-MCGR construct with a 2-year follow-up period.

### 3.5. Complications

**Figure 7:**
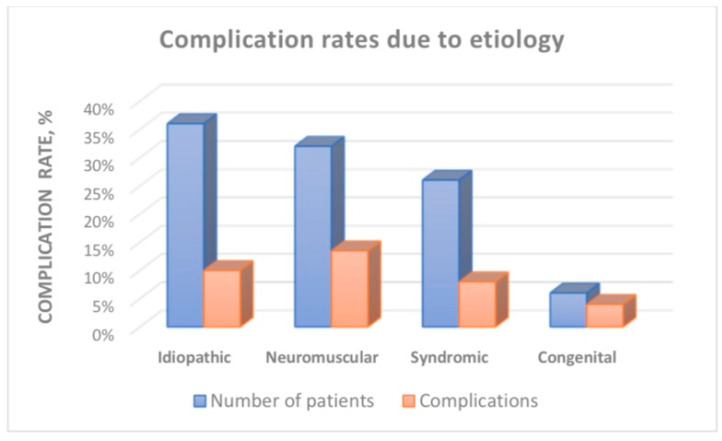
Complication rates in different etiologies.

During the lengthening period, 57 patients developed at least one medical and mechanical complication (35%), and some patients experienced several complications (Table 6). Regarding complications, 33 patients required unplanned surgery (20%). After the final fusion, there were no complications in 48 patients (30%) who converted to PSF during the follow-up period, and no unplanned surgeries were recorded. Figure 7 shows the complication rate in different etiologies of the spinal deformity.

### 3.6. Posterior Spinal Fusion Outcomes

**Figure 8:**
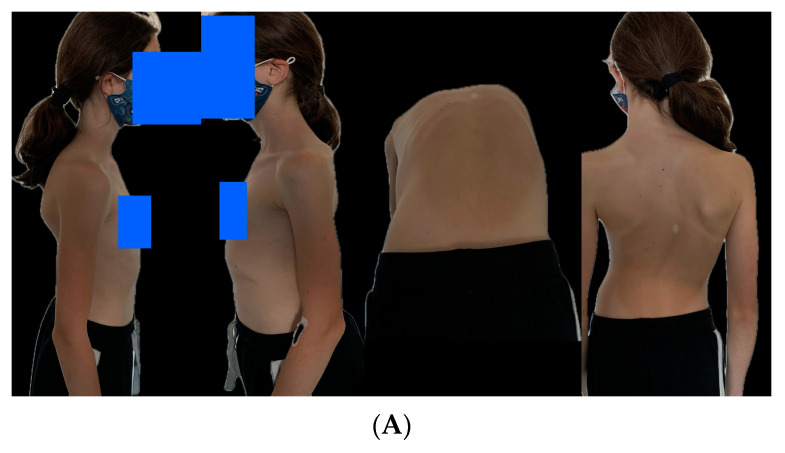

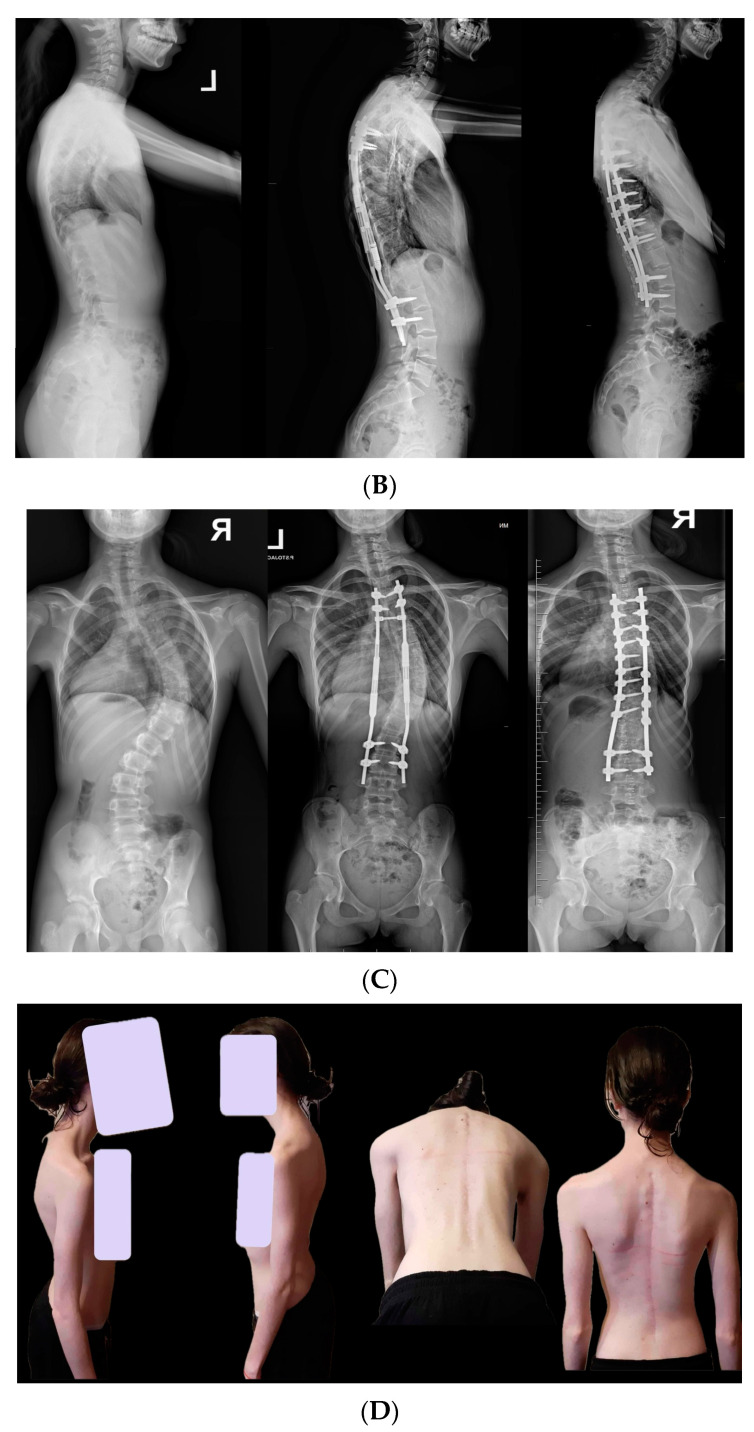
Eight-year-old girl with EOIS (**A**) treated with MCGRs and converted to PSF after a 4-year treatment course without any complications (**B**,**C**). Final follow-up photo (**D**).

The mean age at final fusion was 14.5 years (range: 11–17 years), and the mean follow-up time after PSF was 24 months (range: 13–26 months). Figure 8 presents an 8-year-old girl with early-onset idiopathic scoliosis, treated with MCGRs, who then underwent conversion to PSF after a 4-year treatment course with no complications.

**Figure 9:**
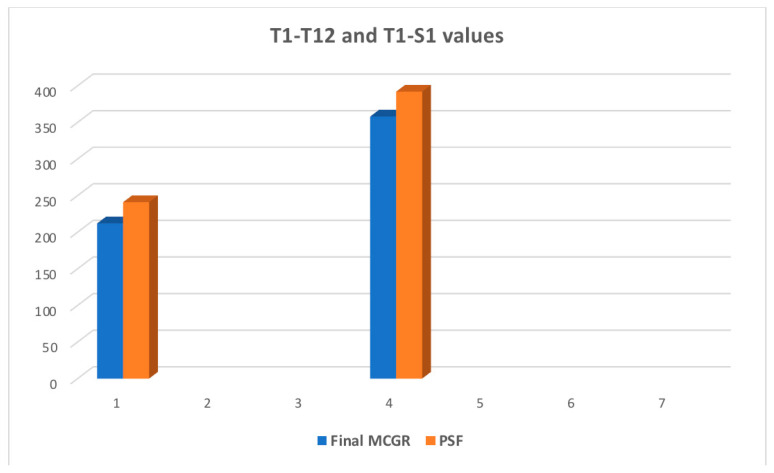
Comparison of the T1–T12 and T1–S1 values after conversion from MCGRs to PSF for 48 patients.

**Figure 10:**
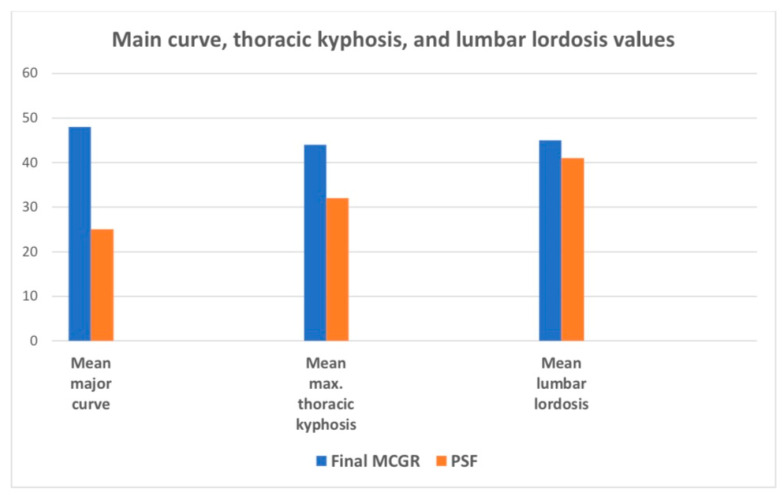
Comparison of the major curve, thoracic kyphosis, and lumbar lordosis values that caused conversion from MCGRs to PSF for 48 patients.

The last follow-up for MCGR patients before conversion to posterior spinal fusion was significantly different based on the mean major curve, thoracic kyphosis, T1–T12, and T1–S1 values, as shown in Figure 9 and Figure 10.

The mean MC pre-conversion to PSF was 48.8° and post-conversion was 25° (*p* < 0.001), while the mean TK improved from 44.5° to 32.5° (*p* < 0.001), the mean T1–T12 improved from 212 to 241 mm (*p* < 0.001), and the mean T1–S1 from improved from 358 to 392 mm (*p* < 0.001), respectively. All data are presented in Table 7.

## 4. Discussion

In our study, we presented the long-term outcomes of patients treated with MCGRs, who were of different ages and etiologies, with 30% of patients having reached the final outcome—conversion of MCGRs to PSF. Knowing from the literature the risk of systemic complications and the potential consequences for health and life resulting from early spinal fusion, more and more attention is being paid to minimal surgical techniques using distraction systems for the treatment of deformations of the growing spine [6]. Since its introduction into widespread use, MCGR technology has shown great promise. However, a growing body of evidence has revealed the potential of this technology to have varied effects on the patient’s body [6,7,8]. EOS is a complex disorder with diverse manifestations and natural histories. Any spinal deformity detected before the age of 5 years is considered an EOS case, with the patient at increased risk for progression and complications secondary to residual growth [3]. For example, EOS may involve the adverse development of breathing problems with age, thus indicating TIS. Diseases that affect the thorax and spine can cause deformity of the spine. When considering the available literature, our data and results confirm that the MCGR method is an effective technique for the treatment of spinal deformities in the youngest patients. Indeed, we were able to achieve satisfactory correction of the main curvature and thoracic kyphosis and obtain adequate spine growth using this technique. During the analysis of patients participating in this study, we obtained radiological data that showed changes in the Cobb angle of the main curvature, the angle of total thoracic kyphosis, and T1–T12 and T1–S1 length measurements in the preoperative period, during the period of continued lengthening of the magnetic rods, and during the postoperative period in patients who underwent conversion to PSF.

The correction of deformities during initial MCGR implantation results in an increase in the length of the T1–T12 and T1–S1 dimensions of the spine, affirming the purpose of using growth-promoting distraction instruments is to facilitate spine growth, especially in the period from implantation to final spinal fusion. This is important for constitutional growth, such as lung development and respiratory efficiency. The available literature regarding the growth rate of the T1–T12 segment of the spine is diverse, ranging from 1.5 to 13.2 mm/year [12,17,23,24,25,26,27,28,29]. The average T1–T12 increase during the observation period in our group of patients was 5.95 mm/year, which is similar to the values obtained by Subramanian et al. [30] and Cheung et al. [16]. Meanwhile, Lebel et al. [14] reported an average growth rate of 0.5 mm/month. In another study, the annual T1–S1 and T1–T12 longitudinal extensions were 8.7 and 4.7 mm/year, respectively [19]. Treatment of severe EOS with single rods has demonstrated an increase in T1–S1 length of 9.4 mm/year and in T1–T12 length of 4.6 mm/year [10], which are comparable to published reports on dual MCGRs. However, in relation to the population of healthy children, the growth rate of the T1–T12 and T1–S1 segments in our patients was slightly lower [5]. We can clearly confirm a positive correlation between the radiological observation period and the height of T1–T12 and T1–S1 segments, so we conclude that a longer treatment time results in an increase in spine height values in the assessed segments. The statistical analysis revealed a significant postoperative increase in the height of the T1–T12 and T1–S1 segments and during the follow-up period during the last MCGR treatment visit (*p* < 0.0001). Treatment with the MCGR system promotes the growth of the spine as the patient grows. However, the time required to achieve sufficient spine growth may be longer than previously understood. Theologis et al. [31] analyzed 1797 pulmonary function test studies of 149 children, and the percent-predicted FEV1 and FVC values for normal children with a T1–T12 height of 22 cm at skeletal maturity were <50%. The authors concluded that these values were concerning and may not be adequate to guarantee that children with early-onset scoliosis who are fused with T1–T12 heights of 22 cm will have an asymptomatic pulmonary status in adulthood [31]. Regarding the T1–T12 and T1–S1 segment results in patients treated with other surgical techniques, a recent systemic review concluded that the T1–T12 growth rate in patients treated with MCGRs was 0.6 mm/month (range: 0.2–1.2 mm/month), with an average follow-up period of 1.5 years [9]. In another systematic review study that compared the growth among growth-friendly systems for scoliosis, the authors indicated that all systems often report values similar to Dimeglio’s T1–S1 spinal growth of 1 cm/year [5]. It should be recognized though that a considerable portion of the reported spinal growth is the result of the initial and final surgical correction and not due to the growth-friendly implant [9]. However, in comparison with the results of the treatment of a large group of patients using the standard growing rod technique [32] with an average follow-up period of 8.2 years, the authors showed an average T1–T12 growth rate of 0.25 mm/month. The differences may be attributed to the length of the follow-up period, the number of repeated operations to distract the instruments, the development of scarring and autofusion, and the “law of diminishing returns” [33,34,35,36]. From this, we can conclude that the ability of the growing system to control deformation as the child grows, distract the rods, and minimize autofusion is very important. In the study by Johnston et al., the authors described that regardless of a thoracic height of ≤18 or >18 cm, with residual curves of >50°, pulmonary function was ominously low in half of the patients, raising doubt about the value of this threshold as an EOS outcome parameter [4]. Early spinal fusion has considerably negative consequences in future life. The pulmonary function in patients who had undergone thoracic spinal fusion for scoliosis prior to the age of 6 continued to decline into adulthood at a rate faster than that of their peers. The majority of these patients had clinically restrictive lung disease, which may be fatal. Alternative treatment strategies should be considered [37]. El Bromboly et al. showed that at a minimum of 5 years of follow-up, distraction-based surgeries increased the thoracic height for patients with EOS to greater than 18 cm in 65% of patients; however, only 48% of congenital patients reached this thoracic height threshold [38].

In a systematic review by Kan et al., the authors concluded that larger thoracic Cobb angles, greater apical vertebral rotation angles, or hypokyphosis were significantly associated with more significant pulmonary impairments in patients with AIS, although the evidence was limited. From a clinical perspective, the results highlight the importance of minimizing three-dimensional spinal deformities to preserve lung function in these patients [39,40]. Johnston et al. indicated that preoperative PFTs are clinically impaired in 19% of AIS patients and correlate significantly with the MT and sagittal plane deformity severity and with PT curve severity to a lesser degree. PFTs do not correlate with the degree of axial deformity [41]. In our study, we showed a significant correction of the Cobb angle from the preoperative period to the final follow-up period at the group level. This correlates well with other MCGR treatment studies [15,27,30,41]. We also found that a significant correction was achieved after surgery after removing the MCGRs and converting the patients to PSF. We were also able to prove that MCGRs control the correction achieved during the primary surgery (MCGR implantation) and allow for an increase in chest dimensions by increasing the growth of the T1–T12 and T1–S1 segments, without significantly changing the thoracic kyphosis. We did not observe a statistically significant worsening of deformities in the coronal plane. During the conversion of MCGRs to PSF, we achieved an increase in the mean correction of the Cobb angle of the main curvature from 48.8° to 25° and an improvement in the mean TK from 44.5° to 32.5°, as well as an increase in the dimensions of T1–T12 and T1–S1 by 29 and 34 mm on average, respectively. We also observed improved coronal deformation by 34%, showing that after MCGRs, the spine has some residual elasticity that can be increased by osteotomy procedures. In our case, the surgical technique of spine correction when replacing MCGRs with fixed instruments always included a wide posterior release—multi-level Ponte osteotomy, often with separation of fused ribs, which are the result of long-term treatment with the MCGR system. In comparison with starting treatment with MCGRs, the mean final coronal correction between the start of MCGR treatment and after undergoing PSF was 70%. The mean T1–T12 spinal height increased by 75 mm, while the mean T1–S1 spinal height increased by 97 mm. Other studies have shown, after the TGR treatment course to conversion to PSF [34], an average of 44% additional correction when proceeding from TGR to the final union, with an average of seven Smith–Petersen osteotomies. Helenius et al. showed an average increase in the T1–T12 segment of 10 mm with a 24% correction of the main curvature during conversion to PSF and an average overall curvature correction from the use of MCGRs to PSF of 51% [36].

Based on our results, it can be concluded that single rods can be as effective and safe as double rods. This is relevant to surgeons as it is not possible to implant double MCGR rods in all patients or all curvatures. Our clinical experience shows that congenital and syndromic cases, which often have short and less flexible curves, are better suited to treatment with single rods. Unfortunately, our patient group contained too few patients with single rods to allow for an objective analysis. The single- and double-rod patient groups were not entirely comparable, mainly in terms of the etiology of EOS. The single-rod group consisted mainly of congenital and syndromic cases, while the two-rod group consisted mainly of idiopathic and neuromuscular cases [42]. At the group level, the radiographic results were comparable between single and double rods, and complication rates were not statistically different.

There are many scientific reports regarding complications resulting from MCGR treatment. Complications are estimated to range from 20% to 70–80% depending on the calculations in various reports. These reports may not fully reflect the actual values, as most of them were performed on a small number of patients [12,14,19,20,21,43,44]. A detailed analysis of complications will be the subject of future study and publication.

### Limitations

Similar to any study, this one also has its limitations. Due to its multicenter nature, the consistency of data reported and analyzed may have varied among the study sites. Although, theoretically, the surgical technique of MCGR implantation was similar, and each surgeon performed it according to their own learned technique. The period of MCGR extension after surgery was performed according to the same protocol but at different time intervals. Moreover, this group of patients represents patients treated with new technology, with conversion to PSF completed in 30% of patients, so it is possible that we observed a learning process. This is also a strength of our study, in that beyond the number of patients involved and long-term follow-up, MCGR treatment was initiated until completion with posterior spinal fusion. The lack of original data on patients’ respiratory functionality is the biggest limitation of this study.

## 5. Conclusions

MCGR treatment allows for an average correction of the curvature by 50% during the period of lengthening, and 70% during PSF, while controlling the deformation and growth of the spine. A statistically significant increase in the T1–T12 and T1–S1 segments was obtained during the observation period. Deformity correction and spine growth were comparable in cases treated with single and double rods. MCGR treatment in EOS carries a risk of complications, resulting in unplanned surgeries in approximately 20% of cases. The incidence of complications and unplanned surgery differed significantly depending on the etiology of the curve. MCGRs limit the number of elective surgeries required to lengthen the instrumentation.

## Figures and Tables

**Table 1:** Demographic data.

Demographic *n* = 161	All Patients *n* = 161
N (%)	161 (100%)
Age in years (SD) at insertion of MCGRs	7.08 (2.32)
Mean (SD) FU, months	32.8 (27.5)
Gender, *n* (%)	
Male	71 (44%)
Female	90 (56%)
Etiology, *n* (%)	
Congenital	10 (6%)
Idiopathic	58 (36%)
Neuromuscular	51 (32%)
Syndromic	42 (26%)
Rod diameter, *n* (%) of rods	
a. 4.5 mm, 5.0 mm	66 (22%)
b. 5.5 mm, 6.0 mm	236 (78%)
Rod length, *n* (%) of rods	
a. 70 mm	114 (38%)
b. 90 mm	188 (62%)
Number of pts in which rods inserted	
a. Single rod	20 (12.5%)
b. Double rods	141 (87.5%)
Patients, *n* (%)	
a. Under six years of age	73 (45%)
b. Over six years of age	88 (55%)
*p*-value (a vs. b)	
MC, *n* (%)	
a. More than 90°	66 (41%)
b. Less than 90°	95 (59%)
*p*-value (a vs. b)	
Number of pts who underwent preop with Halo Gravity Traction before MCGR insertion, *n* (%)	32 (20%)
Number of pts who underwent anterior release before MCGR insertion, *n* (%)	16 (10%)

**Table 2:** Preoperative, postoperative, and FFU radiological outcomes of the coronal and sagittal Cobb angles by subgroup. IS—idiopathic scoliosis, SS—syndromic scoliosis, NS—neuro-muscular scoliosis, CS—congenital scoliosis.

Variable	IS (*n* = 58) A	SS (*n* = 42) B	NS (*n* = 51) C	CS (*n* = 10) D	Total (*n* = 161)	*p*-Value
Mean (SD) age at MCGR implantation in years	7.2 (2.8)	8.3 (2.3)	7.6 (3.1)	8.4 (2.2)	7.08 (2.3)	0.9743
FFU (SD) in months	39.5 (29.2)	34.5 (26.8)	34.5 (28.8)	39.5 (29.8)	32.8 (27.5)	0.9819
Mean (SD) preoperative major curve, degrees	85.3 (22)	82.5 (24)	88.2 (25)	81.9 (19)	86.2 (21)	0.4134
Mean (SD) postoperative major curve, degrees	45.3 (12)	51.2 (16)	44.8 (11)	44.6 (10)	46.9 (14)	0.4323
Mean (SD) major curve at FFU, degrees	43.1 (13)	48.7 (14)	47.7 (15)	45.2 (12)	45.8 (12)	0.5271
Preoperative vs. FFU comparisons	*p* < 0.001	*p* < 0.001	*p* < 0.001	*p* < 0.001	*p* < 0.001	
Mean (SD) preoperative thoracic kyphosis, degrees	55.2 (21)	39.4 (19)	35.3 (17)	47.1 (19)	47.2 (20)	0.4139
Mean (SD) postoperative thoracic kyphosis, degrees	40.1 (18)	38.4 (16)	33.2 (15)	39.3 (14)	47.1 (17)	0.3627
Mean (SD) max. thoracic kyphosis at FFU, degrees	32.8 (16)	36.4 (15)	37.5 (14)	41.2 (14)	44.5 (15)	0.3988
Preoperative vs. FFU comparisons	*p* < 0.001	*p* > 0.05	*p* > 0.05	*p* < 0.001	*p* > 0.05	
Mean (SD) preoperative lumbar lordosis, degrees	45.7 (16)	40.2 (15)	45.8 (14)	41.7 (13)	44.2 (14)	0.8863
Mean (SD) postoperative lumbar lordosis, degrees	41.9 (12)	42.4 (11)	36.9 (12)	37.7 (13)	39 (12)	0.8928
Mean (SD) lumbar lordosis at FFU, degrees	48.9 (13)	38.9 (12)	37.8 (11)	40.2 (12)	45.8 (11)	0.9469
Preoperative vs. FFU comparisons	*p* > 0.05	*p* > 0.05	*p* > 0.05	*p* > 0.05	*p* > 0.05	

**Table 3:** Preoperative, postoperative, and FFU T1–T12 and T1–S1 outcomes by subgroup. IS—idiopathic scoliosis, SS—syndromic scoliosis, NS—neuro-muscular scoliosis, CS—congenital scoliosis.

Variable	IS (*n* = 58) A	SS (*n* = 42) B	NS (*n* = 51) C	CS (*n* = 10) D	Total (*n* = 161)
Mean (SD) preoperative T1–T12 height in mm	138 (34)	158 (38)	165 (41)	142 (38)	166 (36)
Mean (SD) postoperative T1–T12 height in mm	165 (37)	183 (39)	190 (38)	171 (38)	188 (39)
Mean T1–T12 height in mm at FFU	202 (36)	205 (37)	204 (36)	193 (36)	208 (38)
Preoperative vs. FFU comparisons	*p* < 0.001	*p* < 0.001	*p* < 0.001	*p* < 0.001	*p* < 0.001
Postoperative comparisons					
A vs. B	A vs. C	A vs. D	B vs. C	B vs. D	C vs. D
*p* > 0.05	*p* > 0.05	*p* < 0.001	*p* > 0.05	*p* < 0.001	*p* < 0.001
Mean (SD) preoperative T1–S1 height in mm	282 (58)	293 (62)	282 (65)	262 (58)	295 (65)
Mean (SD) postoperative T1–S1 height in mm	317 (55)	328 (58)	315 (58)	297 (54)	328 (63)
Mean (SD) T1–S1 height in mm at FFU	334 (48)	364 (53)	351 (52)	342 (49)	368 (55)
Preoperative vs. FFU comparisons	*p* < 0.001	*p* < 0.001	*p* < 0.001	*p* < 0.001	*p* < 0.001
Postoperative comparisons					
A vs. B	A vs. C	A vs. D	B vs. C	B vs. D	C vs. D
*p* < 0.001	*p* < 0.001	*p* > 0.05	*p* > 0.05	*p* < 0.001	*p* > 0.05

**Table 4:** MCGR lengthening outcomes during follow-up by subgroup. IS—idiopathic scoliosis, SS—syndromic scoliosis, NS—neuro-muscular scoliosis, CS—congenital scoliosis.

Variable	IS (*n* = 58) A	SS (*n* = 42) B	NS (*n* = 51) C	CS (*n* = 10) D	Total (*n* = 161)
Mean (SD) length of distraction phase, months	36.5 (14.2)	33.9 (9.8)	29.7 (11.2)	26.8 (8.8)	37.5 (13.8)
A vs. B *p* > 0.05	A vs. C *p* < 0.001	A vs. D *p* < 0.001	B vs. C *p* > 0.05	B vs. D *p* > 0.05	C vs. D *p* > 0.05
Mean (SD) number of times a patient underwent lengthening per year	6.5 (1.5)	5.5 (2.2)	5.8 (1.2)	6.2 (1.4)	6.7 (1.3)
A vs. B *p* > 0.05	A vs. C *p* > 0.05	A vs. D *p* > 0.05	B vs. C *p* > 0.05	B vs. D *p* > 0.05	C vs. D *p* > 0.05

**Table 5:** MCGR T1–T12 and T1–S1 outcomes during the lengthening period by subgroup. IS—idiopathic scoliosis, SS—syndromic scoliosis, NS—neuro-muscular scoliosis, CS—congenital scoliosis.

Variable	IS (*n* = 58) A	SS (*n* = 42) B	NS (*n* = 51) C	CS (*n* = 10) D	Total (*n* = 161)
Mean (SD) T1–T12 growth in mm/year at FFU	6.2 (1.8)	5.8 (1.2)	5.5 (1.5)	5.2 (1.1)	5.95 (2.2)
A vs. B *p* < 0.001	A vs. C *p* < 0.001	A vs. D *p* < 0.001	B vs. C *p* > 0.05	B vs. D *p* > 0.05	C vs. D *p* < 0.001
Mean (SD) T1–S1 growth in mm/year at FFU	10.8 (3.2)	9.1 (2.8)	9.8 (2.6)	8.8 (2.8)	10.1 (3.4)
A vs. B *p* > 0.05	A vs. C *p* > 0.05	A vs. D *p* < 0.001	B vs. C *p* > 0.05	B vs. D *p* > 0.05	C vs. D *p* < 0.001

**Table 6:** MCGR lengthening complication rates during follow-up by subgroup. IS—idiopathic scoliosis, SS—syndromic scoliosis, NS—neuromuscular scoliosis, CS—congenital scoliosis; some patients experienced several complications.

Complications	IS (*n* = 58)	SS (*n* = 42)	NS (*n* = 51)	CS (*n* = 10)	Total Value
infection	1	1	4	1	7
anchor pull-out	2	2	3	1	8
rod breakage	4	2	4	1	11
pin fracture	1	2	3	0	6
distraction failure	7	3	5	3	18
adding-on	0	1	0	0	1
PJK	1	2	2	1	6
Total	16 (10%)	13 (8%)	21 (13%)	7 (4%)	57 (35%)

**Table 7:** MCGR vs. PSF outcomes for 48 patients.

Variable Total (*n* = 48)	Final MCGR	PSF	*p*-Value
Mean (SD) major curve at FFU, degrees	48.8 (12)	25 (13)	*p* < 0.001
Mean (SD) max. thoracic kyphosis at FFU, degrees	44.5 (15)	32.5 (14)	*p* < 0.001
Mean (SD) lumbar lordosis at FFU, degrees	45.8 (13)	41 (11)	*p* > 0.05
Mean (SD) T1–T12 height in mm at FFU	212 (36)	241 (48)	*p* < 0.001
Mean (SD) T1–S1 height in mm at FFU	358 (61)	392 (45)	*p* < 0.001

## Data Availability

The data are contained within this article.
